# Cerebral amyloid angiopathy impacts neurofibrillary tangle burden and cognition

**DOI:** 10.1093/braincomms/fcae369

**Published:** 2024-11-22

**Authors:** Dana Godrich, Jeremy Pasteris, Eden R Martin, Tatjana Rundek, Gerard Schellenberg, Tatiana Foroud, Jeffery M Vance, Margaret A Pericak-Vance, Michael L Cuccaro, William K Scott, Walter Kukull, Thomas J Montine, Gary W Beecham

**Affiliations:** Dr. John T Macdonald Foundation Department of Human Genetics, Miller School of Medicine, University of Miami, Miami, FL 33136, USA; Dr. John T Macdonald Foundation Department of Human Genetics, Miller School of Medicine, University of Miami, Miami, FL 33136, USA; John P. Hussman Institute for Human Genomics, Miller School of Medicine, University of Miami, Miami, FL 33136, USA; Dr. John T Macdonald Foundation Department of Human Genetics, Miller School of Medicine, University of Miami, Miami, FL 33136, USA; John P. Hussman Institute for Human Genomics, Miller School of Medicine, University of Miami, Miami, FL 33136, USA; Department of Neurology and Evelyn F. McKnight Brain Institute, Miller School of Medicine, University of Miami, Miami, FL 33136, USA; Penn Neurodegeneration Genomics Center, Department of Pathology and Laboratory Medicine, Perelman School of Medicine, University of Pennsylvania, Philadelphia, PA 190104, USA; Department of Medical and Molecular Genetics, Indiana University, Indianapolis, IN 46202, USA; Dr. John T Macdonald Foundation Department of Human Genetics, Miller School of Medicine, University of Miami, Miami, FL 33136, USA; John P. Hussman Institute for Human Genomics, Miller School of Medicine, University of Miami, Miami, FL 33136, USA; Dr. John T Macdonald Foundation Department of Human Genetics, Miller School of Medicine, University of Miami, Miami, FL 33136, USA; John P. Hussman Institute for Human Genomics, Miller School of Medicine, University of Miami, Miami, FL 33136, USA; Dr. John T Macdonald Foundation Department of Human Genetics, Miller School of Medicine, University of Miami, Miami, FL 33136, USA; John P. Hussman Institute for Human Genomics, Miller School of Medicine, University of Miami, Miami, FL 33136, USA; Dr. John T Macdonald Foundation Department of Human Genetics, Miller School of Medicine, University of Miami, Miami, FL 33136, USA; John P. Hussman Institute for Human Genomics, Miller School of Medicine, University of Miami, Miami, FL 33136, USA; Department of Epidemiology, University of Washington, Seattle, WA 351619, USA; Department of Pathology, Stanford University, Stanford, CA 94305, USA; Dr. John T Macdonald Foundation Department of Human Genetics, Miller School of Medicine, University of Miami, Miami, FL 33136, USA; John P. Hussman Institute for Human Genomics, Miller School of Medicine, University of Miami, Miami, FL 33136, USA

**Keywords:** neuropathology, neurodegeneration, Alzheimer’s disease, dementia

## Abstract

Cerebral amyloid angiopathy commonly co-occurs with amyloid β plaques and neurofibrillary degeneration and is proposed to contribute to cognitive impairment. However, the interplay among these pathologic changes of Alzheimer disease is not well understood. Here we replicate and extend findings of a recent study that suggested the association of cerebral amyloid angiopathy and cognitive impairment is mediated by neurofibrillary degeneration. We employed similar approaches but in a larger, clinical-based (as opposed to community-based) set of 4915 autopsied National Alzheimer’s Coordinating Center participants (60% with dementia). Neuropathologic lesions were measured ordinally; longitudinal change in cognition was used to measure cognitive impairment. Statistical analyses included ordinal logistic regression, mediation analyses and extension of models to include presence of *APOE* e4. We show a statistical interaction between cerebral amyloid angiopathy and neuritic plaques that impacts the burden of neurofibrillary tangles. Mediation analyses show that cerebral amyloid angiopathy is associated with cognitive impairment, but only by modifying the impact of neurofibrillary tangles on cognition. We expanded the mediation analysis to include *APOE* e4 and show similar results. Findings indicate that cerebral amyloid angiopathy plays an important role in the burden and impact of neurofibrillary degeneration contributing to cognitive impairment.

## Teaser

Godrich *et al*. assess the role of cerebral amyloid angiopathy and apolipoprotein E on the amyloid, Tau and dementia pathway. Authors confirm that cerebral amyloid angiopathy interacts with amyloid to impact cognition via Tau burden. This holds when accounting for apolipoprotein E; that is, it is not simply a pleiotropic effect.

## Graphical Abstract

**Graphical Abstract:**
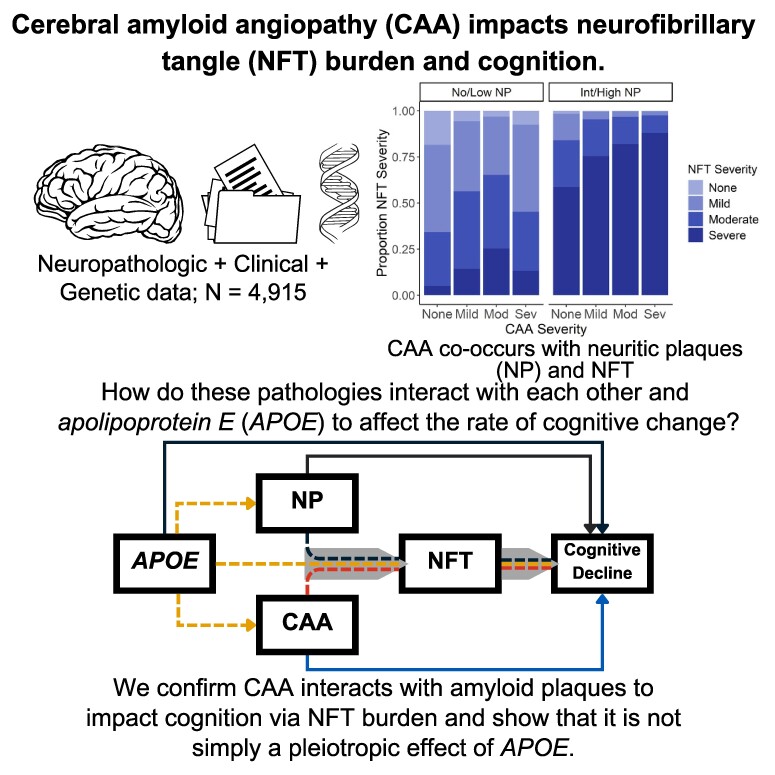

## Introduction

**Figure 1:**
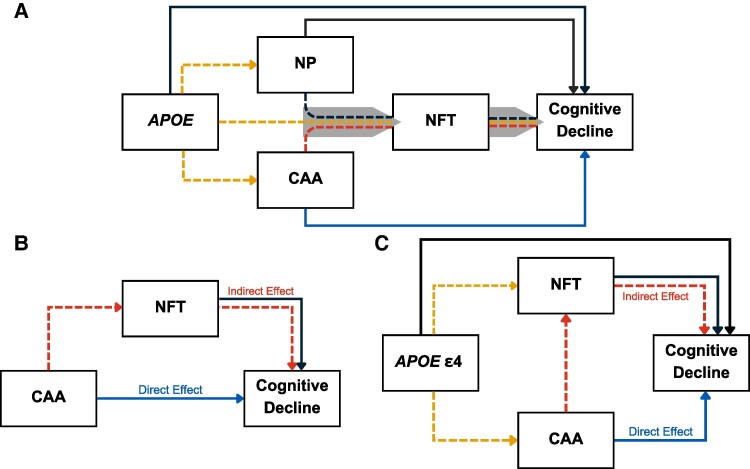
**Illustration of path models. A** illustrates the hypothetical relationship between *APOE*, amyloid (neuritic) plaques, CAA, neurofibrillary tangle (NFT) progression and rate of cognitive change (adapted from Rabin *et al.*). Briefly, *APOE* increases amyloidosis (NP and CAA) while also potentially having other direct effects on NFT and cognition. CAA may have direct effects on cognition but may also operate through NFT indirectly (dashed lines), while also interacting with other effects such as *APOE* and plaques. Operationally, we tested this model through mediation analyses subset by NP severity. **B** and **C** illustrate the mediation analyses. The first (**B**) tests both the direct effect and indirect effect of CAA on change in cognition. The second (**C**) tests the same effects, but also accounts for *APOE* e4 dosage, to assess the role of *APOE* pleiotropic effects. NFT, neurofibrillary tangles; CAA, cerebral amyloid angiopathy; APOE e4, Apolipoprotein E e4 allele.

Over 55 million individuals worldwide are affected by dementia, and Alzheimer disease is the most common form of dementia, accounting for 60–80% of cases.^1^ Patients with Alzheimer disease typically present with two hallmark neuropathologic changes at autopsy: neurofibrillary tangles (NFT) and amyloid plaques.^2-6^ Aggregation of paired helical filament (PHF) Tau forms NFTs, while amyloid neuritic plaques (NP) are the subset of extracellular parenchymal β-amyloid deposits (Aβ) that also harbour dystrophic neurites containing PFH tau and are most closely related to the disease.^7^ These hallmark pathologic changes seldom appear in isolation, often co-occur with hallmark features of Alzheimer disease-related dementias (ADRDs).^8-16^ One such feature is cerebral amyloid angiopathy (CAA). CAA is an amyloidosis of the walls of small arteries, arterioles and capillaries in the brain. CAA commonly co-occurs with the Alzheimer disease neuropathologic changes (ADNC), and the apolipoprotein E (*APOE*) e4 allele is a risk factor of both ADNC and CAA severity.^16-20^ CAA has also been associated with clinically diagnosed dementia, cognitive impairment and functional impairment by us and others.^8-16,21^ It is estimated that 70–98% of individuals with dementia also have CAA.^8,14,21^ While it seems likely that CAA, NP and NFT may work together to impact cognition (Fig. 1A), the exact relationships are unknown.

Rabin *et al.*^22^ recently used statistical modelling to examine the relationship between CAA, ADNC and cognitive decline. They found that CAA did not have a direct impact on cognitive decline, but rather exerted an indirect effect via NFT burden (Fig. 1B). That is, there was more NFT (and with worse cognitive outcomes) in the presence of CAA than without CAA. This mediation effect was only significant in the intermediate/high NP group.

In this study, we sought to replicate and generalize the findings of Rabin *et al.*,^22^ in a set of 4915 individuals from the National Alzheimer’s Coordinating Centers (NACC). This dataset is larger than the Rabin *et al.*^22^ study (*n* = 4915 compared to Rabin *et al.*,^22^  *n* = 1722) and is composed of memory and aging clinic-based participants from across the USA, rather than the community-based ascertainment scheme of Rabin *et al.*^22^ Additionally, while *APOE* e4 is clearly associated with ADNC, CAA and cognition, its role in the CAA and NFT mediation has not been evaluated. To address this gap, we extend analyses to include *APOE*, testing if the effect of CAA on cognition via NFT burden is a pleiotropic effect of *APOE* (Fig. 1A and C).

## Materials and methods

### Study participants and inclusion criteria

This study consists of individuals who consented to inclusion in research and later underwent brain autopsy. Participants were enrolled and assessed clinically through the multisite, nationwide NIH-funded Alzheimer’s Disease Research Centers (ADRC), under a standardized protocol. Data were derived from the Uniform Data Set (UDS) of the NACC, together with the NACC neuropathology data (accessed June 2020).^6^

The UDS consists of participant clinical, medical and demographic data. The neuropathology data includes detailed information on neuropathological features from standardized brain autopsy evaluation. We only retained individuals with age at death (AAD) > 50 years who are not missing relevant pathologic data (detailed in Section 2.2), AAD, education, sex, race and *APOE* e4 (*n* = 4915). Participants were predominantly non-Hispanic white by self-report, both white and black participants were included (Table 1). There were too few participants from other race groups to have adequate statistical power and were thus excluded. All participants (or representatives) provided written informed consent; all protocols and assessments were performed with approval by the appropriate institutional internal review boards.

**Table 1:** Cohort characteristics

	NACC (*n* = 4915)	
	No or low neuritic plaques	Int. or high neuritic plaques
Number of participants, *n* (%)	1710 (35%)	3205 (65%)
Age at baseline, mean (SD)	76.7 (11.3)	75.6 (10.1)
Age at death, mean (SD)	81.7 (12.0)	80.9 (10.3)
Education, mean (SD)	15.5 (3.1)	15.4 (3.1)
Females, *n* (%)	802 (47%)	1517 (47%)
Black participants, *n* (%)	62 (4%)	123 (4%)
Decline in cognition, mean (SD)^a,b^	−1.34 (1.48)	−2.29 (1.49)
Time (years) between baseline and final visits, mean (SD)^a^	4.8 (3.0)	4.6 (2.8)
Number of visits, mean (SD)^a^	5.3 (2.8)	5.0 (2.4)
*APOE* e4, *n* (%)		
0 copies	1293 (76%)	1427 (44%)
1 copy	380 (22%)	1399 (44%)
2 copies	37 (2%)	379 (12%)
Final clinical diagnosis, *n* (%)		
No cognitive impairment	628 (37%)	443 (14%)
Impaired, not MCI	60 (3%)	48 (2%)
MCI	284 (17%)	491 (15%)
Dementia	738 (43%)	2223 (69%)
CAA severity, *n* (%)		
None	1144 (67%)	789 (25%)
Mild	355 (21%)	1077 (34%)
Moderate	158 (9%)	814 (25%)
Severe	53 (3%)	525 (16%)
Braak NFT stage, *n* (%)		
Stage 0	240 (14%)	24 (1%)
Stages I–II	752 (44%)	192 (6%)
Stages III–IV	562 (33%)	584 (18%)
Stages V–VI	156 (9%)	2405 (75%)

NFT, neurofibrillary tangles; CAA, cerebral amyloid angiopathy; APOE e4, Apolipoprotein E e4 allele; MCI, mild cognitive impairment.

^a^Sample size for decline, years-follow-up and number of visits is 3571 (1244 with no or low NP and 2327 with intermediate or high NP).

^b^Decline based on Mini-Mental State Exam (MMSE) and cross-walked Montreal Cognitive Assessment (MOCA).

### Neuropathology phenotypes and variable coding

Relevant lesions include NFTs, NPs and CAA, extracted from the neuropathology subset of the NACC dataset.^6^ NFT burden was derived from Braak stage and was then condensed into a four-level ordinal variable (B score in NIA-AA guidelines: 0, neurofibrillary degeneration not present; B1, Braak I or II; B2, Braak III or IV; B3, Braak V or VI; missing otherwise).^2-6^ NP burden was derived from the density of neocortical neuritic plaques (CERAD) score as a level-level ordinal variable (C score: C0, no NP; C1, sparse NP; C2, moderate NP; C3, frequent NP; missing otherwise).^2-6^ CAA burden was also a four-level ordinal variable (none, mild CAA, moderate CAA, severe CAA, missing otherwise).^6,23,24^  *APOE* genotype was reported by the ADRC, and was analysed as the number of *APOE* e4 alleles (0, 1, or 2).^6^

### Clinical outcomes

Neurocognitive impairment was assessed using the Mini-Mental State Examination (MMSE), and the Montreal Cognitive Assessment (MoCA).^25-27^ MMSE and MoCA are primarily screening tools for gross changes in cognition, which limits its precision for small changes in cognition. However, the tool is widely available, and thus maximizes sample size. MoCA was first cross-walked to the MMSE using an equipercentile approach.^28^ Cognition was then estimated using a linear mixed model. MMSE and cross-walked MoCA were treated as outcome variables, with time since first assessment (in years) as the independent variable, with mixed effects for slope and intercept grouped by individual. The output of this model is individual estimates of slope (rate) of cognitive decline, using all available MMSE or MoCA assessments. Age and sex were not included in the linear mixed model but were included in subsequent models (below). The approach is only valid for individuals with two or more MMSE/MoCA assessments (*n* = 3571).

### Statistical analysis and models

Statistical analyses were conducted in R version 4.2.2.^29^ Kendall’s tau correlation between primary variables was assessed.

### Association analyses

Two primary models were used to test the effects of CAA and NPs on NFTs: one that tests main effects only (Model 1) and another that tests main and interaction effects (Model 2). Model fitting was performed with ordinal logistic regression and models were adjusted for age at death, years of education, sex, race and copies of *APOE* e4. Model selection was performed on four models: (i) main effects only; (ii) main and CAA by NP interaction, (iii) main and CAA by *APOE* e4 interaction; and (iv) main, CAA by NP and CAA by *APOE* e4 interactions. We assessed model fit using Nagelkerke pseudo-R2 estimates, Akaike Information Criteria (AIC), and likelihood ratio tests. Additional analyses to assess CAA, NPs and NFT include adjustments for baseline cognition (MMSE), assessment of *APOE* e2 and reported ethnicity (see Supplementary material).

### Mediation analyses

We used mediation analyses to assess the relationships among relevant variables. Mediation analysis involves assessing multiple regression models and observing how an effect size changes in the presence or absence of another (mediating) variable. If the effect does not change, there is a direct effect, and there is no mediation. If the effect does change in the presence of the mediating variable, there is an indirect effect and the variable is said to be mediated. We assessed direct and indirect effects using structural equation modelling in the Lavaan package in R.^30^ Lavaan’s latent variable approach has the advantage of being able to appropriately handle ordinal variables. Ordinal endogenous variables (e.g. Braak and CAA) are declared as such in lavaan’s ‘sem’ function, at which point lavaan switches to a weighted least-squares approach, where the diagonally weighted least squares is used to estimate model parameters, and the full weight matrix is used to compute robust standard errors. Two primary models were considered. The first model tests the mediation of NFT burden on CAA’s effect on cognition, where CAA burden is the independent (exogenous) variable, rate of cognitive change is an endogenous variable, and NFT burden an endogenous (ordinal) mediator. The second model incorporates *APOE* e4, testing if NFT burden and CAA mediate the effect of *APOE* e4 on cognition. In this model, *APOE* e4 is the exogenous variable, CAA burden and NFT burden are both mediators and endogenous, ordinal variables, and rate of cognitive change is an endogenous variable. Both models were stratified by NP burden. Additional mediation analyses include assessment of *APOE* e2 and assessments of AD/RD copathology (see Supplementary material).

## Results

### Cohort characteristics

There were 4915 eligible individuals for this study (Table 1, Supplementary Table 1). Most had dementia (60%) or MCI (15.8%), but some were cognitively intact (21.8%) or impaired, but not MCI (2.2%) (Table 1). Neuropathological co-morbidity was common in the dataset, with 56% of individuals exhibiting co-occurring ADNC and CAA. The dataset showed some differences to the previous study,^22^ with the NACC participants having younger at baseline and death, higher proportion male, more *APOE* e4, and more severe NFT, more NP burden and less CAA (Supplementary Table 2).

### Statistical modelling of CAA and ADNC

**Figure 2:**
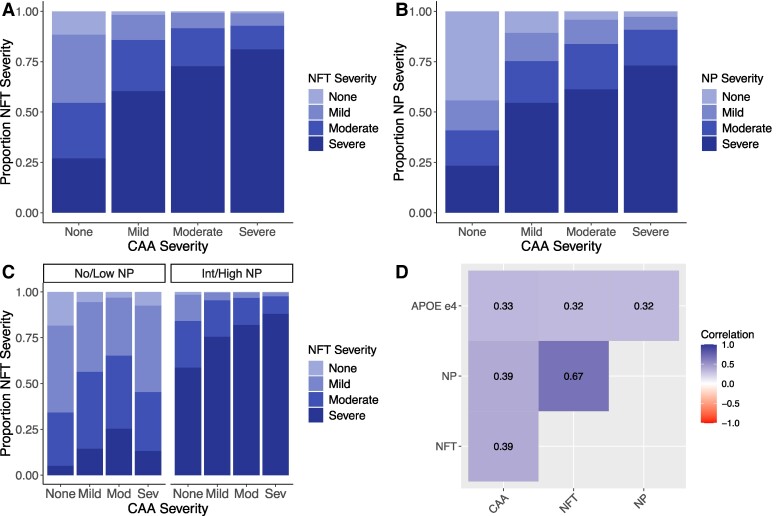
**Correlation and trends of neurofibrillary tangles and neuritic plaque levels by CAA burden.** (**A**) Bar plot of NFT burden by CAA burden. (**B**) Bar plot of NP burden by CAA burden. (**C**) NFT burden by CAA burden, grouped into no/low and intermediate/high NP groups. (**D**) Correlation (Kendall’s τ) between lesions (CAA, NFT, NP) and *APOE* e4. NFT, neurofibrillary tangles; NP, neuritic plaques; CAA, cerebral amyloid angiopathy; APOE e4, Apolipoprotein E e4 allele.

CAA severity increases with the pathologic burden of NPs and NFTs (Fig. 2A–C). NFT and NP are highly correlated (rho = 0.75) and are also moderately correlated with CAA (rho = 0.45) (Fig. 2D). *APOE* e4 dosage was also moderately correlated with the NFT and NP (rho = 0.35) and CAA (rho = 0.36) (Fig. 2D).

NP score and CAA were associated with NFT burden in the main effect model (Supplementary Fig. 1). In the interaction model, the main effect of CAA on NFT burden was reduced, and interaction terms for CAA by NP were associated (Supplementary Fig. 2). In the previous models, we noted the significant association of *APOE* e4 on NFTs, so we tested an additional model with an *APOE* e4 by CAA interaction (Supplementary Fig. 3). None of the *APOE* e4 by CAA interactions were significantly associated with the NFT outcome (Supplementary Fig. 3). When assessing model fit, the CAA by NP interaction model showed the best fit (AIC: 7511; Nagelkerke pseudo-*R*^2^: 0.594), and a statistically significant improvement over the main effects only model (*P* = 0.00056, using an *F*-test) (Supplementary Table 3). Inclusion of baseline MMSE did not appreciably impact the association between CAA and NFT (Supplementary Table 4). Assessment of *APOE* e2 showed a strong protective effect on CAA (one copy of e2, OR = 0.295; two copies OR = 0.191); the association persisted while accounting for presence of NP and NFT (Supplementary Table 5). Inclusion of self-reported ethnicity (Hispanic, not-Hispanic) in the model as a covariate did not appreciably alter the association (Supplementary Table 6). Ethnicity was not associated with NFT (OR = 1.01; *P* = 0.97), and CAA remained associated with NFT. The magnitude of effect of severe CAA on NFT did however increase (OR = 1.26, *P* = 3.8 × 10^−7^ without accounting for ethnicity; OR = 2.23, *P* = 5.9 × 10^−9^ in the model with ethnicity).

### CAA mediation analyses

Because of the above-noted interaction between CAA and NP, and to replicate Rabin *et al.*,^22^ the dataset was stratified by NP burden for mediation analyses. The no/low NP group (*n* = 1205) had less severe NFTs and CAA than the int/high NP group (*n* = 2251).

In the no/low NP group, CAA had no direct effect on rate of cognitive change (*P* = 0.235) but had a nominal indirect effect (Est = −0.386, *P* = 0.029) (Table 2). Additionally, in the no/low NP group, we observed a nominal direct effect of NFT on cognitive change (Est = −1.307, *P* = 0.024) (Table 2). A sensitivity analysis considering dementia status suggests the nominal indirect effect may be confined to the subset of no/low NP with dementia or MCI (Supplementary Text S1). In the int/high NP group, CAA did not directly affect rate of cognitive change but rather was indirectly associated with cognitive change through NFT burden (Table 2; Est = −0.131, *P* < 0.001). NFT burden directly contributed to worse cognitive change (Table 2; Est = −0.397, *P* < 0.001). In both NP groups, CAA was directly associated with NFT burden (Table 2). Together, these findings replicate the Rabin *et al.*^22^ study for the int/high NP group. Follow-up analyses show that the CAA/NFT mediation in the int/high NP subset persists, even when including other neuropathologies as covariates in the structural equation models (Supplementary Tables 7–9). Specifically, the indirect effect of CAA and NFT on cognition is at least nominally significant when accounting for Lewy bodies, hippocampal sclerosis, vascular brain injury and arteriolosclerosis, with effect estimates consistently showing a reduction in cognition (faster decline). Similarly, in the int/high NP subset, the indirect effect is significant in a model including all pathologies (Est = −0.133, *P* = 0.004) (Supplementary Table 9).

**Table 2:** Summary of mediation analysis results

	Model 1 (without *APOE* e4)				Model 2 (with *APOE* e4)			
	Low/No NP		Int/High NP		Low/No NP		Int/High NP	
	Effect	*P*	Effect	*P*	Effect	*P*	Effect	*P*
Direct effects								
CAA → NFT	**0.296**	**<0.001**	**0.329**	**<0.001**	**0**.**195**	**<0**.**001**	**0**.**295**	**<0**.**001**
CAA → cognition	0.223	0.235	−0.018	0.635	−1.098	0.069	−0.020	0.565
NFT → cognition	−1.307	0.024	**−0.397**	**<0.001**	−1.050	0.064	**−0**.**369**	**<0**.**001**
*APOE* e4 → CAA					**0**.**745**	**<0**.**001**	**0**.**454**	**<0**.**001**
*APOE* e4 → NFT					**0**.**479**	**<0**.**001**	**0**.**253**	**<0**.**001**
*APOE* e4 → cognition					1.151	0.019	−0.034	0.459
Indirect effects								
CAA → NFT → cognition	−0.386	0.029	**−0.131**	**<0.001**	−0.205	0.071	**−0**.**109**	**<0**.**001**
*APOE* e4 → NFT → cognition					−0.503	0.076	**−0**.**093**	**<0**.**001**
*APOE* e4 → CAA → cognition					−0.817	0.074	−0.009	0.565
*APOE* e4 → CAA → NFT → cognition					−0.153	0.073	**−0**.**049**	**<0**.**001**

Bold values highlight statistically significant values (*P* < 0.01). ‘Cognition’ refers to the longitudinal change in MMSE or cross-walked MOCA scores, using a mixed effects model. A negative value for cognition indicates decreasing performance over time.

### Mediation and *APOE*

We extended the mediation analyses to assess the impact of *APOE* e4 on the relationships between CAA, NFT and impairment (Fig. 1C). By including *APOE* e4 as an independent variable in the mediation models, we test whether direct or indirect effects of CAA and NFT are better explained by a shared relationship to *APOE* e4. The results were similar to the initial mediation analyses (Table 2). In the model with *APOE* e4 count as an independent variable, the no/low NP group showed no direct or indirect effect of CAA on cognition, though the indirect effect was approaching nominal significance (Est = −0.205, *P* = 0.071). In the int/high NP group, the effect of the CAA on cognition via NFT burden remained statistically significant, though the magnitude did drop (Est = −0.109, *P* < 0.001). This indicates that the mediation effect of NFT burden is not simply explained by its correlation with *APOE* e4 (Table 2). Both CAA and NFT played mediating roles in statistically linking *APOE* e4 to worse cognitive change (Table 2). Additional analyses with *APOE* e2 count as a covariate also show the indirect effect of CAA on cognition (Supplementary Table 10). The analyses also reflect the protective effect of APOE e2, but with inconsistent *P*-values (likely due to low statistical power due to the low frequency of e2; allele frequency = 0.06).

## Discussion

Here we investigated the impact of CAA on the Alzheimer disease neuropathological landscape and on rate of cognitive change.^22^ First, we showed a positive correlation between CAA and Alzheimer disease neuropathologic changes. We then showed that CAA did not have an independent effect on NFT burden, but rather had a strong interactive effect with NP burden. The mediation analyses suggest that CAA burden does not impact cognition directly but operates through NFT burden. We see this most clearly in the group with intermediate to high NP burden—an observation consistent with previous reports.^22^ However, we also observed the indirect effect in the no/low NP group, albeit with reduced confidence—an observation inconsistent with previous reports. The reason for this inconsistency is unclear but may be due to statistical power. The no/low group on average has less cognitive change (mean rate of change = −1.34) than the int/high group (−2.29) (Table 1), and disease effects may be more inconsistent for early-stage disease. This would reduce effect estimates and statistical power. This hypothesis is supported by our sensitivity analysis (Supplementary Text S1), where we observed a nominal indirect effect of CAA and NFT on cognition within the subset of no/low NP diagnosed with dementia or MCI.

We investigated the impact of *APOE* e4 on these relationships. One alternate hypothesis is that CAA does not have a true indirect effect but is simply a proxy for some pleiotropic effect of *APOE*. That is, *APOE* could lead to other unmeasured neurological changes (apart from CAA) that impact NFT burden and cognition. Our results, however, do not support this hypothesis. In the model, comparison analyses with the CAA by NP interaction were more informative than a model including *APOE* e4. The CAA model achieved the best fit and better explained the variability in NFT burden. Similarly, in the mediation analyses, we still observed the indirect effect of CAA, even while accounting for the effect of *APOE*. Together, these results suggest that, while *APOE* e4 has its own effects on NFT and cognition, CAA is capturing additional information about NFT and cognition that *APOE* e4 alone is not capturing.

This study has its limitations. ADRC ascertainment is biased towards those with Alzheimer disease or more significant memory impairment, so individuals with normal cognition or mild impairment may be underrepresented. As such, our study may not generalize well to mild pathological changes and mild changes in cognition. While MMSE and MOCA have their strengths, there are available more robust approaches for harmonizing multiple assessment batteries^31^ and for assessing longitudinal cognitive progression. Future efforts could include these more robust measures, and perhaps, investigation of specific cognitive domains. Finally, we note that these data do not well represent the diversity that exists in the general population, notably African American and Hispanic individuals, as the rate of participation in autopsy programmes from these populations tends to be low.

While this study shows a statistical relationship between CAA and NFT, the precise biological nature of that relationship remains unclear. It is not clear, for example, if the true relationship is moderation, mediation, or some of both. The most parsimonious hypotheses are that damage due to CAA makes the brain more susceptible to NFT formation (mediation), or to increased damage from NFT (moderation), perhaps due to oxidative stress.^32^ A second, indirect, possibility is that processes leading to CAA are also leading to increased NFT burden. For example, the differential accumulation of Aβ proteoforms in CAA versus brain parenchyma could signal a pathophysiologic process important to NFT burden. Another hypothesis is that it is due to incomplete modelling of NP. If NP is not modelled correctly, any residual NP association with NFT could be ‘picked up’ by the correlated CAA. We think this last explanation unlikely though, given that Rabin *et al.*^22^ used different (quantitative) measures of CAA, NP and NFT and different cognitive measures yet reached similar conclusions.^22^

Together with this previous work, our study shows an important role for CAA in Alzheimer disease pathology and dementia. Our analyses of *APOE* suggest that this is not solely a pleiotropic effect of *APOE*. This suggests that CAA may have implications for clinical progression and treatment, and as such should be subject to further study.

## Supplementary Material

fcae369_Supplementary_Data

## Data Availability

Primary data are available through qualified access from the National Alzheimer’s Coordinating Center (https://naccdata.org/).
