# Supplementary material for: Cerebral amyloid angiopathy impacts neurofibrillary tangle burden and cognition
Source: Brain Commun. 2024 Nov 22;6(6):fcae369. doi: 10.1093/braincomms/fcae369 (PMC11581998; doi:10.1093/braincomms/fcae369)
Supplement: fcae369_Supplementary_Data [file fcae369_supplementary_data.docx]

**Supplementary** **Materials.**

**Supplementary Text**

**Supplemental Text 1.** Sensitivity Analysis of CAA Mediation

**Supplementary Tables**

**Supplemental Table 1.** Sample Inclusion Criteria

**Supplemental Table 2.** Cohort characteristics of ROSMAP study and NACC study

**Supplemental Table 3.** Comparison of regression models (independent and interaction models)

**Supplemental Table 4.** Adjusting regression models to include baseline MMSE

**Supplemental Table 5.** Effect of *APOE* e2 on CAA

**Supplemental Table 6.** Inclusion of Self-Reported Ethnicity

**Supplemental Table 7.** Copathology mediation models in intermediate to high NP group

**Supplemental Table 8.** Copathology mediation models in no to low NP group

**Supplemental Table 9:** Mediation models with all copathologies

**Supplemental Table 10.** Summary of mediation analysis results modeled with *APOE* e2

**Supplementary Figures**

**Supplemental Figure 1.** Forest plot of independent regression model

**Supplemental Figure 2.** Forest plot of regression model with CAA by NP interaction

**Supplemental Figure 3.** Forest plot of regression model with CAA by *APOE* interaction

**References.**

**Supplemental Text 1. Sensitivity Analysis of CAA Mediation**

One key finding of both our study and Rabin et al^1^ is that the indirect effect of CAA on cognition is inconsistent (or possibly non-existent) when amyloid plaques are minimal (Low/No NP). The reason for this finding is unclear. One hypothesis is that as a disease-modifying process (progression, decline) rather than a disease-causing process (risk), the effect is best seen among a predominantly case set, rather than a mix of cases and cognitive controls. If this is true, we would not see the CAA, NFT, cognition effect in a dataset with predominantly cognitively intact, low-disease individuals, but we would when assessing a dataset with more disease. Simply put, you cannot observe a progression effect when there is very little progression in the dataset.

To test this, we repeated the CAA mediation analyses in the Low/No NP group but split by cognitive status (cognitively normal in one group; demented/MCI in another). Results show that the direct effect of CAA on NFT remained in both groups. However, the nominal indirect effect was only observed in the dementia/MCI group.

|  | | Model 1 (without *APOE* e4) | | | | | | | |
| --- | --- | --- | --- | --- | --- | --- | --- | --- | --- |
|  | | Low/No NP  ALL (N = 1,205) | | Low/No NP  CN/Impaired (N = 341) | | Low/No NP  Dementia/MCI (N = 903) | | Int/High NP  ALL (N = 2,251) | |
|  | | Effect | P | Effect | P | Effect | P | Effect | P |
|  | CAA 🡪 NFT | **0.296** | **<0.001** | **0.181** | **0.047** | **0.315** | **<0.001** | **0.329** | **<0.001** |
|  | CAA 🡪 Cognition | 0.223 | 0.235 | -0.004 | 0.903 | 0.252 | 0.225 | -0.018 | 0.635 |
|  | NFT 🡪 Cognition | -1.307 | 0.024 | 0.025 | 0.773 | -1.352 | 0.028 | **-0.397** | **<0.001** |
|  | CAA 🡪 NFT 🡪 Cognition | -0.386 | 0.029 | 0.005 | 0.795 | -0.426 | 0.035 | **-0.131** | **<0.001** |

Bold values highlight statistically significant values (*P* < 0.01).

“Cognition” refers to the longitudinal change in MMSE or cross-walked MOCA scores, using a mixed effects model. A negative value for cognition indicates decreasing performance over time.

**Supplemental Table 1. Sample Inclusion criteria**

| 5,958 autopsied participants 🡪 Remove 70 participants with AAD less than 50 🡪 |
| --- |
| 5,888 remain 🡪 Remove 140 participants missing CAA information 🡪 |
| 5,748 remain 🡪 Remove 95 participants missing NFT or NP information 🡪 |
| 5,635 remain 🡪 Remove 113 participants missing race information 🡪 |
| 5,540 remain 🡪 Remove 625 missing *APOE* genotype 🡪 |
| 4,915 remain |

**Supplemental Table 2** **Cohort characteristics of ROSMAP study^1^ and NACC study.**

|  | ROSMAP | NACC |
| --- | --- | --- |
| Number of participants, N | 1,722 | 4,915 |
| Age at baseline, mean (SD) | 80.2 (7.1) | 76.0 (10.5) |
| Age at death, mean (SD) | 89.5 (6.7) | 81.2 (10.9) |
| Education, mean years (SD) | 16.2 (3.5) | 15.4 (8.6) |
| Females, *N* (%) | 1,169 (67.9) | 2,319 (47.2) |
| White participants, *N* (%) | 1,636 (95.0) | 4,730 (96.2) |
| *APOE* e4 positive, *N* (%) | 439 (25.5) | 2,196 (44.7) |
| Final clinical diagnosis, *N* (%) |  |  |
| No cognitive impairment | 549 (31.9) | 1,071 (21.8) |
| Impaired, not MCI | NA | 108 (2.2) |
| MCI | 395 (22.9) | 775 (15.8) |
| Dementia | 778 (45.2) | 2,961 (60.2) |
| CAA severity, *N* (%) |  |  |
| None | 391 (22.7) | 1,933 (39.3) |
| Mild | 710 (41.2) | 1,432 (29.1) |
| Moderate | 389 (22.6) | 972 (19.8) |
| Severe | 232 (13.5) | 578 (11.8) |
| CERAD NP score, *N* (%) |  |  |
| None | 405 (23.5) | 1,067 (21.7) |
| Mild | 138 (8.0) | 643 (13.1) |
| Moderate | 605 (35.1) | 958 (19.5) |
| Severe | 574 (33.3) | 2,247 (45.7) |
| Braak NFT stage, *N* (%) |  |  |
| Stage 0 | 20 (1.2) | 264 (5.4) |
| Stage I-II | 267 (15.5) | 944 (19.2) |
| Stage III-IV | 965 (56.0) | 1,146 (23.3) |
| Stage V-VI | 470 (27.3) | 2,561 (52.1) |

*Abbreviations: APOE e4, Apolipoprotein E* ***e****4 allele; CAA, cerebral amyloid angiopathy; CERAD, Consortium to Establish a Registry for Alzheimer’s Disease; MCI, mild cognitive impairment; NACC, National Alzheimer Coordinating Center; NFT, neurofibrillary tangle burden; NP, neuritic plaques; ROSMAP, Religious Orders Study and Memory and Aging Project; SD, standard deviation.*

**Supplemental Table 3.** **Independent and interaction model comparisons**

| **(A)** | **Model** | | **R^2^** | **AIC** |
| --- | --- | --- | --- | --- |
|  | 1 | NFT ~ covars + *APOE* e4 + CAA + NP | 0.591 | 7522 |
|  | 2 | NFT ~ covars + *APOE* e4 + CAA + NP + CAA×NP | 0.594 | 7511 |
|  | 3 | NFT ~ covars + *APOE* e4 + CAA + NP + CAA×*APOE* e4 | 0.592 | 7531 |
|  | 4 | NFT ~ covars + *APOE* e4 + CAA + NP + CAA×NP + CAA×*APOE* e4 | 0.595 | 7519 |
|  | 5 | NFT ~ covars + *APOE* e4 + CAA + NP + *APOE* e4 ×NP | 0.593 | 7540 |
|  | 6 | NFT ~ covars + *APOE* e4 + CAA + NP + *APOE* e4 ×NP×CAA | 0.599 | 7546 |

| **(B)** | **Test** | **N dof** | ***LR* Stat** | ***P(Chi)*** |
| --- | --- | --- | --- | --- |
|  | 2 vs. 1 | 9 | 29.4 | 0.00056 |
|  | 3 vs. 1 | 6 | 2.90 | 0.821 |
|  | 4 vs. 1 | 15 | 32.6 | 0.00054 |
|  | 5 vs. 1 | 6 | 16.3 | 0.012 |
|  | 6 vs. 1 | 37 | 71.8 | 0.00052 |
|  | 4 vs. 2 | 6 | 3.16 | 0.788 |
|  | 5 vs. 2 | 3 | 13.7 | 0.003 |
|  | 6 vs. 2 | 31 | 55.5 | 0.045 |

**(A)** The reduced (independent effects) model and four interaction models we evaluated: (1) reduced, (2) CAA×NP, (3) CAA×*APOE* e4, (4) both CAA×NP and CAA×*APOE* e4, (5) *APOE* e4×NP, and (6) *APOE* e4×NP×CAA, each adjusted by the same covariates (AAD, sex, years of education, and race). When assessing model fit, the CAA×NP model (2) had a low AIC and high Nagelkerke pseudo-*R^2^* compared to the independent model (1), CAA×*APOE* e4 model (3), and double interaction model (4). Even though the three-way interaction model (6) showed a higher Nagelkerke pseudo-*R^2^*, the AIC is worse. **(B)** When comparing models using F-tests, we accepted the CAA×NP model over the other models. Although there is a nominal improvement in fit from model 2 to model 6 (p=0.045), the benefit is overshadowed by the high number of DFs being used, so the AIC is worse.

*Abbreviations: NFT, neurofibrillary tangles; NP, neuritic plaques; CAA, cerebral amyloid angiopathy; APOE e4, Apolipoprotein E e4 allele; Covars, covariates; AIC, Akaike information criterion; N dof, number of degrees of freedom; LR Stat, likelihood ratio statistic.*

**Supplemental Table 4** **Adjusting regression models to include baseline MMSE**

|  | **Model 1**  (no adjustment for baseline MMSE) | | **Model 2**  (adjusting for baseline MMSE) | |
| --- | --- | --- | --- | --- |
|  | OR | P value | OR | P value |
| Sex | 1.03 | 8.2×10^-1^ | 1.04 | 6.0×10^-1^ |
| AAD | 1.01 | 2.9×10^-12^ | 1.04 | 2.8×10^-19^ |
| Education | 0.997 | 6.3×10^-1^ | 1.00 | 9.2×10^-1^ |
| Black race | 0.992 | 3.1×10^-1^ | 0.992 | 3.2×10^-1^ |
| Baseline MMSE | - | - | 0.921 | 1.1×10^-20^ |
| *APOE* e4 – 1 copy | 1.61 | 7.6×10^-8^ | 1.57 | 4.0×10^-7^ |
| *APOE* e4 – 2 copies | 3.31 | 5.0×10^-9^ | 3.17 | 2.4×10^-8^ |
| CAA – mild | 1.63 | 4.4×10^-7^ | 1.67 | 1.7×10^-7^ |
| CAA – moderate | 2.25 | 9.5×10^-12^ | 2.10 | 6.6×10^-10^ |
| CAA – severe | 1.26 | 3.8×10^-7^ | 2.12 | 3.3×10^-6^ |
| NP – mild | 3.48 | 4.3×10^-25^ | 3.44 | 1.6×10^-24^ |
| NP – moderate | 10.51 | 6.3×10^-83^ | 10.09 | 3.8×10^-79^ |
| NP – severe | 90.72 | 1.5×10^-245^ | 77.96 | 6.3×10^-224^ |

Using a sample set with participants with both clinical and neuropathologic data *(N =* 3,456), we tested a similar model to that in Supplementary Figure 1 (NFT ~ sex + AAD + education + race + *APOE* e4 + CAA + NP). Model 1 (without adjusting for baseline MMSE) had OR of similar magnitude and *P*-values of similar significance to Supplementary Figure 1. Model 2 adjusted for baseline MMSE score, which was significant in the model. However, adjusting for baseline MMSE score did not meaningfully alter the other variables in the model.

*Abbreviations: AAD, age at death; MMSE, Mini-Mental State Examination; NFT, neurofibrillary tangles; NP, neuritic plaques; CAA, cerebral amyloid angiopathy; APOE e4, Apolipoprotein E e4 allele.*

**Supplemental Table 5. Effect of *APOE* e2 on CAA**

|  | **Model 1**  CAA ~ covars + *APOE* e2 | | **Model 2**  CAA ~ covars + *APOE* e2 + NP + NFT | |
| --- | --- | --- | --- | --- |
|  | OR | *P* | OR | *P* |
| Sex | 0.855 | 4.5×10^-3^ | 0.810 | 2.3×10^-4^ |
| AAD | 1.010 | 5.9×10^-7^ | 1.01 | 8.7×10^-8^ |
| Education | 0.979 | 1.3×10^-2^ | 0.979 | 2.2×10^-2^ |
| Black race | 0.934 | 6.2×10^-1^ | 0.934 | 6.3×10^-1^ |
| *APOE* e2 – 1 copy | 0.295 | 6.2×10^-107^ | 0.501 | 1.5×10^-31^ |
| *APOE* e2 – 2 copies | 0.191 | 6.5×10^-5^ | 0.568 | 2.0×10^-1^ |
| NP | - | - | 1.63 | 1.4×10^-42^ |
| NFT | - | - | 1.65 | 1.6×10^-26^ |

*Abbreviations: AAD, age at death; APOE e4, Apolipoprotein E e4 allele; NFT, neurofibrillary tangles; NP, neuritic plaques; CAA, cerebral amyloid angiopathy.*

**Supplemental Table 6. Inclusion of Self-Reported Ethnicity**

|  | **Model 1**  (N = 4,915) | | **Model 2**  (N = 4,897) | | **Model 3**  (N = 4,752) | |
| --- | --- | --- | --- | --- | --- | --- |
|  | OR | P value | OR | P value | OR | P value |
| Sex | 1.03 | 8.2×10^-1^ | 1.03 | 6.9×10^-1^ | 1.03 | 6.4×10^-1^ |
| AAD | **1.01** | **2.9×10^-12^** | **1.04** | **6.5×10^-29^** | **1.04** | **1.1×10^-28^** |
| Education | 0.997 | 6.3×10^-1^ | 0.999 | 9.1×10^-1^ | 0.994 | 6.1×10^-1^ |
| Race | 0.992 | 3.1×10^-1^ | 0.939 | 7.1×10^-1^ | 0.911 | 5.9×10^-1^ |
| Ethnicity | **-** | **-** | 1.01 | 9.7×10^-1^ | - | - |
| NP – mild | **3.48** | **4.3×10^-25^** | **3.25** | **1.2×10^-30^** | **3.20** | **2.8×10^-29^** |
| NP – moderate | **10.51** | **6.3×10^-83^** | **9.07** | **1.3×10^-103^** | **9.14** | **2.8×10^-101^** |
| NP – severe | **90.72** | **1.5×10^-245^** | **97.0** | **4.9×10^-357^** | **101.0** | **3.3×10^-350^** |
| CAA – mild | **1.63** | **4.4×10^-7^** | **1.63** | **2.4×10^-9^** | **1.62** | **5.4×10^-9^** |
| CAA – moderate | **2.25** | **9.5×10^-12^** | **2.21** | **1.3×10^-14^** | **2.17** | **1.3×10^-13^** |
| CAA – severe | **1.26** | **3.8×10^-7^** | **2.23** | **5.9×10^-9^** | **2.12** | **6.6×10^-8^** |
| *APOE* e4 – 1 copy | **1.61** | **7.6×10^-8^** | **1.63** | **4.2×10^-11^** | **1.63** | **7.6×10^-11^** |
| *APOE* e4 – 2 copies | **3.31** | **5.0×10^-9^** | **3.75** | **6.0×10^-14^** | **3.75** | **1.2×10^-13^** |

*Abbreviations: AAD, age at death; APOE e4, Apolipoprotein E e4 allele; NP, neuritic plaques; CAA, cerebral amyloid angiopathy.*

**Supplemental Table 7. Copathology** **mediation models in intermediate to high NP group**

|  | **Original model** | | **LB model** | | **HS model** | | **VBI model** | | **Arteriolosclerosis model** | |
| --- | --- | --- | --- | --- | --- | --- | --- | --- | --- | --- |
|  | Int/High NP  (N = 2,327) | | Int/High NP  (N =2,222) | | Int/High NP  (N =2,259) | | Int/High NP  (N =2,007) | | Int/High NP  (N =2,102) | |
|  | Effect | P | Effect | P | Effect | P | Effect | P | Effect | P |
| **Direct Effects** | | | | | | | | | | |
| CAA 🡪 NFT | **0.329** | **<0.001** | **0.325** | **<0.001** | **0.327** | **<0.001** | **0.295** | **<0.001** | **0.355** | **<0.001** |
| Copathology 🡪 NFT | -- | -- | 0.008 | 0.730 | 0.227 | 0.015 | 0.012 | 0.741 | -0.015 | 0.662 |
| CAA 🡪 Cognition | -0.018 | 0.635 | -0.013 | 0.738 | -0.017 | 0.713 | -0.033 | 0.610 | 0.017 | 0.638 |
| Copathology 🡪 Cognition | -- | -- | -0.031 | 0.203 | -0.098 | 0.261 | 0.083 | 0.015 | -0.049 | 0.115 |
| NFT 🡪 Cognition | **-0.397** | **<0.001** | **-0.394** | **<0.001** | **-0.384** | **0.002** | -0.404 | 0.045 | **-0.394** | **<0.001** |
| **Indirect Effects** | | | | | | | | | | |
| CAA 🡪 NFT 🡪 Cognition | **-0.131** | **<0.001** | **-0.141** | **<0.001** | **-0.125** | **0.002** | -0.119 | 0.040 | **-0.143** | **<0.001** |

*“Original model” includes structural equations, with sex and AAD as covariates. “LB”, “HS”, “VBI”, and “Arteriolosclerosis” models include Lewy bodies, hippocampal sclerosis, vascular brain injury, and arteriolosclerosis as covariates, respectively.*

*Bold values highlight statistically significant values (P < 0.01).*

*“Cognition” refers to the longitudinal change in MMSE or cross-walked MOCA scores, using a mixed effects model. A negative value for cognition indicates decreasing performance over time.*

*Abbreviations: MMSE, Mini-Mental State Examination; MOCA, Montreal Cognitive Assessment; NFT, neurofibrillary tangles; NP, neuritic plaques; CAA, cerebral amyloid angiopathy; APOE e4, Apolipoprotein E e4 allele; LB, Lewy bodies; HS, hippocampal sclerosis; VBI, vascular brain injury; Art., arteriolosclerosis.*

**Supplemental Table 8. Copathology mediation models in no to low NP group**

|  | **Original model** | | **LB model** | | **HS model** | | **VBI model** | | **Arteriolosclerosis model** | |
| --- | --- | --- | --- | --- | --- | --- | --- | --- | --- | --- |
|  | No/Low NP  (N =1,244) | | No/Low NP  (N =1,208) | | No/Low NP  (N =1,221) | | No/Low NP  (N =1,123) | | No/Low NP  (N =1,102) | |
|  | Effect | P | Effect | P | Effect | P | Effect | P | Effect | P |
| **Direct Effects** | | | | | | | | | | |
| CAA 🡪 NFT | **0.296** | **<0.001** | **0.276** | **<0.001** | **0.297** | **<0.001** | **0.295** | **<0.001** | **0.217** | **<0.001** |
| Copathology 🡪 NFT | -- | -- | **0.174** | **<0.001** | -0.121 | 0.244 | **0.174** | **<0.001** | 0.016 | 0.692 |
| CAA 🡪 Cognition | 0.223 | 0.235 | 0.216 | 0.158 | 0.216 | 0.158 | -0.099 | 0.549 | -0.100 | 0.404 |
| Copathology 🡪 Cognition | -- | -- | 0.190 | 0.057 | **-1.157** | **<0.001** | 0.013 | 0.801 | **-0.136** | **0.006** |
| NFT 🡪 Cognition | -1.307 | 0.024 | **-1.295** | **0.008** | -1.272 | 0.021 | -0.200 | 0.698 | 0.061 | 0.902 |
| **Indirect Effects** | | | | | | | | | | |
| CAA 🡪 NFT 🡪 Cognition | -0.386 | 0.029 | -0.357 | 0.014 | -0.378 | 0.028 | -0.059 | 0.711 | 0.013 | 0.904 |

*“Original model” includes structural equations, with sex and AAD as covariates. “LB”, “HS”, “VBI”, and “Arteriolosclerosis” models include Lewy bodies, hippocampal sclerosis, vascular brain injury, and arteriolosclerosis as covariates, respectively.*

*Bold values highlight statistically significant values (P < 0.01).*

*“Cognition” refers to the longitudinal change in MMSE or cross-walked MOCA scores, using a mixed effects model. A negative value for cognition indicates decreasing performance over time.*

*Abbreviations: MMSE, Mini-Mental State Examination; MOCA, Montreal Cognitive Assessment; NFT, neurofibrillary tangles; NP, neuritic plaques; CAA, cerebral amyloid angiopathy; APOE e4, Apolipoprotein E e4 allele; LB, Lewy bodies; HS, hippocampal sclerosis; VBI, vascular brain injury; Art., arteriolosclerosis.*

**Supplemental Table 9: Mediation models with all copathologies**

|  | **Original model** | | | | **Copathology model** | | | |
| --- | --- | --- | --- | --- | --- | --- | --- | --- |
|  | Low/No NP  (N = 1,244) | | Int/High NP  (N = 2,327) | | Low/No NP  (N = 976) | | Int/High NP  (N = 1,723) | |
|  | Effect | P | Effect | P | Effect | P | Effect | P |
| **Direct Effects** | | | | | | | | |
| CAA 🡪 NFT | **0.296** | **<0.001** | **0.329** | **<0.001** | **0.198** | **<0.001** | **0.331** | **<0.001** |
| LB 🡪 NFT | - | - | - | - | **0.146** | **<0.001** | 0.003 | 0.921 |
| HS 🡪 NFT | - | - | - | - | -0.081 | 0.531 | 0.206 | 0.062 |
| VBI 🡪 NFT | - | - | - | - | -0.011 | 0.877 | -0.001 | 0.972 |
| Arte. 🡪 NFT | - | - | - | - | 0.018 | 0.682 | -0.046 | 0.245 |
| CAA 🡪 Cognition | 0.223 | 0.235 | -0.018 | 0.635 | -0.072 | 0.543 | 0.013 | 0.808 |
| LB 🡪 Cognition | - | - | - | - | -0.033 | 0.690 | -0.023 | 0.401 |
| HS 🡪 Cognition | - | - | - | - | **-1.01** | **<0.001** | -0.127 | 0.206 |
| VBI 🡪 Cognition | - | - | - | - | 0.060 | 0.180 | 0.096 | 0.018 |
| Arte. 🡪 Cognition | - | - | - | - | **-0.152** | **0.005** | -0.078 | 0.048 |
| NFT 🡪 Cognition | -1.307 | 0.024 | **-0.397** | **<0.001** | 0.030 | 0.953 | **-0.401** | **0.002** |
| **Indirect Effect** | | | | | | | | |
| CAA 🡪 NFT 🡪 Cognition | -0.386 | 0.029 | **-0.131** | **<0.001** | 0.006 | 0.955 | **-0.133** | **0.004** |

*“Original model” includes structural equations, with sex and AAD as covariates. “Copathology model” also includes Lewy bodies, hippocampal sclerosis, vascular brain injury, and arteriolosclerosis as covariates.*

*Bold values highlight statistically significant values (P < 0.01).*

*“Cognition” refers to the longitudinal change in MMSE or cross-walked MOCA scores, using a mixed effects model. A negative value for cognition indicates decreasing performance over time.*

*Abbreviations: MMSE, Mini-Mental State Examination; MOCA, Montreal Cognitive Assessment; NFT, neurofibrillary tangles; NP, neuritic plaques; CAA, cerebral amyloid angiopathy; APOE e4, Apolipoprotein E e4 allele; LB, Lewy bodies; HS, hippocampal sclerosis; VBI, vascular brain injury; Art., arteriolosclerosis.*

**Supplemental Table 10. Summary of mediation analysis results modeled with *APOE* e2**

|  | *APOE* e2 Model | | | |
| --- | --- | --- | --- | --- |
|  | Low/No NP  (N = 1,244) | | Int/High NP  (N = 2,327) | |
|  | Effect | *P* | Effect | *P* |
| **Direct Effects** | | | | |
| CAA 🡪 NFT | **0.285** | **<0.001** | **0.346** | **<0.001** |
| CAA 🡪 Cognition | -0.095 | 0.876 | -0.033 | 0.354 |
| NFT 🡪 Cognition | -0.144 | 0.788 | **-0.369** | **<0.001** |
| *APOE* e2 🡪 CAA | -0.190 | 0.035 | 0.036 | 0.642 |
| *APOE* e2 🡪 NFT | -0.092 | 0.199 | **-0.260** | **0.009** |
| *APOE* e2 🡪 Cognition | 0.131 | 0.382 | 0.241 | 0.028 |
| **Indirect Effects** | | | | |
| CAA 🡪 NFT 🡪 Cognition | -0.041 | 0.790 | **-0.128** | **<0.001** |
| *APOE* e2 🡪 NFT 🡪 Cognition | 0.013 | 0.846 | **0.096** | **0.010** |
| *APOE* e2 🡪 CAA 🡪 Cognition | 0.018 | 0.894 | -0.001 | 0.687 |
| *APOE* e2 🡪 CAA 🡪 NFT 🡪 Cognition | 0.008 | 0.823 | -0.005 | 0.643 |

*Bold values highlight statistically significant values (P < 0.01).*

*“Cognition” refers to the longitudinal change in MMSE or cross-walked MOCA scores, using a mixed effects model. A negative value for cognition indicates decreasing performance over time.*

*Abbreviations: MMSE, Mini-Mental State Examination; MOCA, Montreal Cognitive Assessment; NFT, neurofibrillary tangles; NP, neuritic plaques; CAA, cerebral amyloid angiopathy; APOE e2, Apolipoprotein E e2 allele.*

**Supplemental Figure 1** **Forest plot of independent regression model.** Summary statistics from the model evaluating the independent effects of cerebral amyloid angiopathy (CAA) and neuritic plaque (NP) burden on NFT severity using ordinal logistic regression. (Model: NFT ~ Age at Death + Education + Sex + Race + *APOE* e4 count + NP + CAA). CAA and NP severity, as well as *APOE* e4 count, are significantly (*P* < 0.05) associated with severity of neurofibrillary tangles. The sample size used in this ordinal logistic regression model was 4,915.

*Abbreviations*: *AAD, age at death; APOE e4, Apolipoprotein E e4 allele; CAA, cerebral amyloid angiopathy; CI, confidence interval; EDUC, years of education; NFT, neurofibrillary tangles; NP, neuritic plaques; OR, odds ratio*.

**Supplemental Figure 2** **Forest plot of regression model with CAA by NP interaction.** Summary statistics from model evaluating the interaction between cerebral amyloid angiopathy (CAA) and neuritic plaque (NP) burden using ordinal logistic regression. (Model: NFT ~ Age at Death + Education + Sex + Race + *APOE* e4 count + NP + CAA + NP×CAA). The interaction terms between CAA and NP are mostly significantly (*P* < 0.05) associated with severity of neurofibrillary tangles, especially the terms that include moderate and severe plaque pathology. CAA does not show a significant independent effect on neurofibrillary tangle severity, but neuritic plaques do. The sample size used in this ordinal logistic regression model was 4,915.

*Abbreviations*: *AAD, age at death; APOE e4, Apolipoprotein E e4 allele; CAA, cerebral amyloid angiopathy; CI, confidence interval; EDUC, years of education; NFT, neurofibrillary tangles; NP, neuritic plaques; OR, odds ratio*.

**Supplemental Figure 3 Forest plot of regression model with CAA by *APOE* interaction.** Summary statistics from model evaluating the interaction effects of cerebral amyloid angiopathy (CAA) and *APOE* e4 count on NFT severity using ordinal logistic regression. (Model: NFT ~ Age at Death + Education + Sex + Race + *APOE* e4 + NP + CAA + CAA×*APOE* e4). CAA and NP severity, as well as *APOE* e4 count, are significantly (*P* < 0.05) associated with severity of neurofibrillary tangles. However, all the interaction terms between CAA and *APOE* e4 count lack significant associations to NFT burden. The sample size used in this ordinal logistic regression model was 4,915.

*Abbreviations*: *AAD, age at death; APOE e4, Apolipoprotein E e4 allele; CAA, cerebral amyloid angiopathy; CI, confidence interval; EDUC, years of education; NFT, neurofibrillary tangles; NP, neuritic plaques; OR, odds ratio*.

**References**

1. Rabin JS, Nichols E, La Joie R, et al. Cerebral amyloid angiopathy interacts with neuritic amyloid plaques to promote tau and cognitive decline. *Brain*. 2022;145(8):2823-2833.
